# Supplementary material for: Combinatorial single-cell profiling of major chromatin types with MAbID
Source: Nat Methods. 2023 Dec 4;21(1):72–82. doi: 10.1038/s41592-023-02090-9 (PMC10776404; doi:10.1038/s41592-023-02090-9)
Supplement: Supplementary file 1 — Supplementary Tables 1 and 2. [file 41592_2023_2090_MOESM1_ESM.pdf]

nature methods

Article https://doi.org/10.1038/s41592-023-02090-9

Combinatorial single-cell profiling of major chromatin types with MAbID

In the format provided by the authors and unedited

Supplementary Table 1 - Antibody list

List of the antibodies used, either as 1) primary antibodies in combination with secondary antibody-DNA conjugates, 2) to generate secondary antibody-DNA conjugates, 3) to generate primary antibody-DNA conjugates or 4) commercial antibody-fluorophore conjugates against BM cell surface markers.

**Primary antibodies used in combination with secondary antibody-DNA conjugates**	**Target**	**Host**	**Type**	**Clonality**	**Vendor**	**Catalog number**	**LOT numbers**	**Optimal working concentration**	**Application**
Anti-Lamin B1 antibody - Nuclear Envelope Marker	Lamin B1	Rabbit	Primary	Polyclonal	Abcam	ab16048	GR3398319-7, GR3369248-1	10 μg/mL	Combined with anti-rabbit IgG conjugate
Histone H3K9me2 antibody (pAb)	H3K9me2	Rabbit	Primary	Polyclonal	Active Motif	39041	39239, 34718002	1 in 100 dilution	Combined with anti-rabbit IgG conjugate
H3K9me3 Recombinant Rabbit Monoclonal Antibody (RM389)	H3K9me3	Rabbit	Primary	Monoclonal	Invitrogen	MA5-33395	WI3388337, WH3388337	5 μg/mL	Combined with anti-rabbit IgG conjugate
Tri-methyl-histone-H3 (Lys27) Rabbit mAb	H3K27me3	Rabbit	Primary	Polyclonal	Cell Signaling Technologies	9733S	16,19	1 in 200 dilution	Combined with anti-rabbit IgG conjugate
Recombinant Anti-Histone H3 (tri methyl K27) antibody [EPR18607] - BSA and Azide free	H3K27me3	Rabbit	Primary	Monoclonal	Abcam	ab222481	GR3256223-6, GR3256223-1	5 μg/mL	Combined with anti-rabbit IgG conjugate
Anti-Trimethyl-Histone H3 (Lys36) antibody, clone RM155	H3K36me3	Rabbit	Primary	Monoclonal	RevMab	31-1051-00	T-04-02948	0.5-1 μg/mL	Combined with anti-rabbit IgG conjugate
Histone H3 trimethyl K36 antibody	H3K36me3	Mouse	Primary	Monoclonal	In-house by Hiroshi Kimura	CM333	-	2 μg/mL	Combined with anti-mouse IgG conjugate
H3K4me3 Monoclonal Antibody (G.532.8)	H3K4me3	Rabbit	Primary	Monoclonal	Invitrogen	MA5-11199	WG3341041, WH334779	121-242 ng/mL	Combined with anti-rabbit IgG conjugate
Histone H3 (mono methyl K4) antibody	H3K4me1	Rabbit	Primary	Polyclonal	Abcam	ab8895	GR3402097-1	10 μg/mL	Combined with anti-rabbit IgG conjugate
Recombinant Anti-Histone H3 (acetyl K27) antibody [EP16602]	H3K27ac	Rabbit	Primary	Monoclonal	Abcam	ab177178	GR3202987-6, GR3202987-19	7 μg/mL	Combined with anti-rabbit IgG conjugate
Recombinant Anti-RNA polymerase II CTD repeat YSPTSPS (phospho S5) antibody [3E8]	Pol II CTD Ser5P	Rat	Primary	Monoclonal	Abcam	ab252852	GR3302510-1, GR33352497-1	5 μg/mL	Combined with anti-rat IgG conjugate
CTCF Antibody	CTCF	Rabbit	Primary	Polyclonal	Diagenode	C15410210	A2354-0010	10 μg/mL	Combined with anti-rabbit IgG conjugate
SUZ12 (D39F6) XP® Rabbit mAb	SUZ12	Rabbit	Primary	Monoclonal	Cell Signaling Technologies	3737S	8	1 in 50 dilution	Combined with anti-rabbit IgG conjugate
Histone H3 Antibody	Histone H3	Sheep	Primary	Polyclonal	Novus Biologicals	NB100-747	p60919	25 μg/mL	Combined with anti-sheep IgG conjugate

**Secondary antibody-DNA conjugates**	**Target**	**Host**	**Type**	**Clonality**	**Vendor**	**Catalog number**	**LOT numbers**	**Optimal working concentration**	**Application**
AffiniPure Goat Anti-Rabbit IgG (H+L)	Rabbit IgG	Goat	Secondary	Polyclonal	Jackson ImmunoResearch	111-005-144	147466	2 μg/mL	Seconday antibody-conjugate
AffiniPure Donkey Anti-Rabbit IgG (H+L)	Rabbit IgG	Donkey	Secondary	Polyclonal	Jackson ImmunoResearch	711-005-152	156033	2 μg/mL	Seconday antibody-conjugate
AffiniPure Donkey Anti-Mouse IgG (H+L)	Mouse IgG	Donkey	Secondary	Polyclonal	Jackson ImmunoResearch	715-005-150	155934	2 μg/mL	Seconday antibody-conjugate
AffiniPure Donkey Anti-Rat IgG (H+L)	Rat IgG	Donkey	Secondary	Polyclonal	Jackson ImmunoResearch	712-005-150	154663	2 μg/mL	Seconday antibody-conjugate
AffiniPure Donkey Anti-Sheep IgG (H+L)	Sheep IgG	Donkey	Secondary	Polyclonal	Jackson ImmunoResearch	713-005-147	150929	2 μg/mL	Seconday antibody-conjugate

**Primary antibody-DNA conjugates**	**Target**	**Host**	**Type**	**Clonality**	**Vendor**	**Catalog number**	**LOT numbers**	**Optimal working concentration**	**Application**
Anti-Lamin B1 antibody - Nuclear Envelope Marker	Lamin B1	Rabbit	Primary	Polyclonal	Abcam	ab16048	GR3398319-I, GR3417466-I, GR3459550	20-35 μg/mL	Primary antibody-conjugate
H3K9me3 Recombinant Rabbit Monoclonal Antibody (RM389)	H3K9me3	Rabbit	Primary	Monoclonal	Invitrogen	MA5-33395	WI3388337, XC3525394	10-25 μg/mL	Primary antibody-conjugate
Recombinant Anti-Histone H3 (tri methyl K27) antibody [EPR18607] - BSA and Azide free	H3K27me3	Rabbit	Primary	Monoclonal	Abcam	ab222481	GR3256223-6, 1024409-1	20-30 μg/mL	Primary antibody-conjugate
Anti-Trimethyl-Histone H3 (Lys36) antibody, clone RM155	H3K36me3	Rabbit	Primary	Monoclonal	RevMab	31-1051-00	T-04-02948, T-04-029-48, V-08-05440	10-20 μg/mL	Primary antibody-conjugate
H3K4me3 Monoclonal Antibody (G.532.8)	H3K4me3	Rabbit	Primary	Monoclonal	Invitrogen	MA5-11199	WH3347791, WG3341041, WI3395412, XA3475081, XJ3741121, XL3785741	10 μg/mL	Primary antibody-conjugate
Histone H3 (mono methyl K4) antibody	H3K4me1	Rabbit	Primary	Polyclonal	Abcam	ab8895	GR3402097-I, GR3426435-2, GR208955-12	10-15 μg/mL	Primary antibody-conjugate
Recombinant Anti-Histone H3 (acetyl K27) antibody [EP16602]	H3K27ac	Rabbit	Primary	Monoclonal	Abcam	ab177178	GR3202987-6, GR3202987-19, GR3202987-29	20-30 μg/mL	Primary antibody-conjugate

**Commercial antibody-fluorophore conjugates against BM cell surface markers**	**Target**	**Host**	**Type**	**Clonality**	**Vendor**	**Catalog number**	**LOT numbers**	**Optimal working concentration**	**Application**
Alexa Fluor® 647 anti-mouse Ly-6G/Ly-6C (Gr-1) Antibody	GR-1 (Granulocytes)	Rat	Primary	Monoclonal	Biolegend	108418	B287274	62.5 ng/mL	BM cell surface marker FACS sorting
Brilliant Violet 421™ anti-mouse CD19 Antibody	CD19 (T cells)	Rat	Primary	Monoclonal	Biolegend	115549	B328655	2 μg/mL	BM cell surface marker FACS sorting
PE anti-mouse TER-119/Erythroid Cells Antibody	TER119 (Erythroblasts)	Rat	Primary	Monoclonal	Biolegend	116208	B311713	500 ng/mL	BM cell surface marker FACS sorting
APC/Cyanine7 anti-mouse CD3 Antibody	CD3 (B cells)	Rat	Primary	Monoclonal	Biolegend	100222	B324939	2 μg/mL	BM cell surface marker FACS sorting
Alexa Fluor® 488 anti-mouse NK-1.1 Antibody	NK-1.1 (NK cells)	Mouse	Primary	Monoclonal	Biolegend	108718	B316543	1.25 μg/mL	BM cell surface marker FACS sorting

Supplementary Table 2 - Significant differentially expressed genes in mouse bone marrow scMAbID dataset

List of significant differentially expressed genes identified in the mouse bone marrow scMAbID dataset, using the enhancer chromatin types as input.

Genes were identified using the FindAllMarkers function of the Signac[71] package, genes were filtered on Pvalues smaller than 0.001.

'Cluster/cell type' indicates in which cell type the gene was differentially expressed.

'Found in referenced dataset' indicates whether the gene was also significantly detected for the same cell type in publicly available sortChIC data[45].

Gene name	p-value	Average_log2FoldChange	Pct.1	Pct.2	Adjusted p-value	Cluster/cell type	Found in referenced dataset
Tbc1d8	1,55E-06	2,2510167	0,043	0,011	3,38E-02	Granulocytes	Yes
Oosp2	2,67E-06	4,6416563	0,012	0	5,81E-02	Granulocytes	No
Gart	3,67E-06	2,1836661	0,028	0,005	7,99E-02	Granulocytes	No
Gm11992	1,83E-05	4,3081496	0,01	0	3,98E-01	Granulocytes	No
Zbtb34	1,90E-05	2,1644372	0,02	0,003	4,15E-01	Granulocytes	No
9830107B12Rik	3,30E-05	3,6963185	0,012	0,001	7,19E-01	Granulocytes	No
Asah1	3,33E-05	3,016767	0,012	0,001	7,24E-01	Granulocytes	No
Trem1	3,34E-05	3,265632	0,012	0,001	7,27E-01	Granulocytes	Yes
Bank1	2,34E-11	1,8495017	0,065	0,014	5,09E-07	B cells	Yes
Col14a1	2,18E-10	2,7567348	0,041	0,005	4,76E-06	B cells	No
Mtss1	5,83E-08	1,722192	0,061	0,018	1,27E-03	B cells	No
Ebf1	1,64E-07	1,2549994	0,074	0,027	3,58E-03	B cells	Yes
Pag1	2,78E-06	1,3271173	0,045	0,013	6,05E-02	B cells	No
Lgr6	7,29E-06	2,1136925	0,033	0,008	1,59E-01	B cells	Yes
Myo1d	7,74E-06	1,114468	0,05	0,017	1,69E-01	B cells	Yes
Fhod3	1,02E-05	0,9666419	0,055	0,02	2,21E-01	B cells	No
Pax7	1,17E-05	1,5381679	0,03	0,007	2,55E-01	B cells	No
A930011G23Rik	1,25E-05	0,9213962	0,068	0,029	2,72E-01	B cells	No
Fam134b	1,58E-05	1,7119589	0,038	0,011	3,43E-01	B cells	No
Gucd1	2,17E-05	1,0954169	0,044	0,015	4,73E-01	B cells	No
Ppp1r16b	2,46E-05	1,3696933	0,039	0,012	5,36E-01	B cells	Yes
Gm10643	4,40E-05	2,9860347	0,012	0,001	9,59E-01	B cells	No
Neurl4	4,40E-05	2,8502142	0,012	0,001	9,59E-01	B cells	No
Ocm	1,50E-05	3,7998005	0,01	0	3,27E-01	Erythroblasts	No
Chtop	1,50E-05	3,4747157	0,01	0	3,27E-01	Erythroblasts	No
Rpl12	2,72E-05	3,2735221	0,012	0,001	5,91E-01	Erythroblasts	No
Osgin1	2,73E-05	3,0458316	0,012	0,001	5,93E-01	Erythroblasts	No
Rbm6	2,75E-05	2,4137255	0,026	0,005	5,99E-01	Erythroblasts	No
Slc22a20	2,95E-05	3,4704053	0,014	0,001	6,42E-01	Erythroblasts	No
Ubxn6	2,07E-06	3,963233	0,011	0	4,50E-02	NK cells	No
C1qtnf2	1,47E-05	3,6183201	0,017	0,001	3,19E-01	NK cells	No
S100a10	1,48E-05	3,3948529	0,017	0,001	3,22E-01	NK cells	Yes
Ccr2	1,48E-05	3,1675248	0,017	0,001	3,22E-01	NK cells	Yes
Tcf3	2,72E-05	2,5619361	0,02	0,002	5,93E-01	NK cells	No
Rita1	3,00E-05	3,6740546	0,014	0,001	6,53E-01	NK cells	No
Bcl11b	4,73E-07	2,5816693	0,047	0,01	1,03E-02	T cells	Yes
Grp	1,18E-06	4,1627662	0,012	0	2,56E-02	T cells	No
Mrps7	1,18E-06	4,0456803	0,012	0	2,56E-02	T cells	No
Pgpep1l	8,39E-06	3,228583	0,017	0,001	1,83E-01	T cells	Yes
Pdcd10	8,47E-06	2,6626206	0,017	0,001	1,85E-01	T cells	No
Usp20	8,58E-06	2,6725256	0,017	0,001	1,87E-01	T cells	No
Nadk	1,50E-05	3,2024678	0,02	0,002	3,28E-01	T cells	Yes
Hmg20a	1,71E-05	2,6316801	0,026	0,004	3,73E-01	T cells	No
1700049G17Rik	3,15E-05	3,2834989	0,012	0	6,87E-01	T cells	No
Nucb1	3,17E-05	2,798757	0,012	0	6,90E-01	T cells	No
Trnp1	3,17E-05	3,0145601	0,012	0	6,90E-01	T cells	No
Snupn	3,17E-05	2,7084929	0,012	0	6,90E-01	T cells	No
Azin2	4,09E-05	3,6133861	0,017	0,002	8,92E-01	T cells	No
Pik3cd	4,36E-05	2,2518009	0,026	0,005	9,50E-01	T cells	Yes
