# Supplementary material for: Combinatorial single-cell profiling of major chromatin types with MAbID
Source: Nat Methods. 2023 Dec 4;21(1):72–82. doi: 10.1038/s41592-023-02090-9 (PMC10776404; doi:10.1038/s41592-023-02090-9)
Supplement: Supplementary file 2 — Reporting Summary [file 41592_2023_2090_MOESM2_ESM.pdf]

nature portfolio

Corresponding author(s): Jop Kind

Last updated by author(s): Sep 20, 2023

Reporting Summary

Nature Portfolio wishes to improve the reproducibility of the work that we publish. This form provides structure for consistency and transparency in reporting. For further information on Nature Portfolio policies, see our Editorial Policies and the Editorial Policy Checklist.

Statistics

For all statistical analyses, confirm that the following items are present in the figure legend, table legend, main text, or Methods section.

n/a	Confirmed	
☐	☒	The exact sample size (n) for each experimental group/condition, given as a discrete number and unit of measurement
☐	☒	A statement on whether measurements were taken from distinct samples or whether the same sample was measured repeatedly
☐	☒	The statistical test(s) used AND whether they are one- or two-sided<br>*Only common tests should be described solely by name; describe more complex techniques in the Methods section.*
☒	☐	A description of all covariates tested
☒	☐	A description of any assumptions or corrections, such as tests of normality and adjustment for multiple comparisons
☐	☒	A full description of the statistical parameters including central tendency (e.g. means) or other basic estimates (e.g. regression coefficient) AND variation (e.g. standard deviation) or associated estimates of uncertainty (e.g. confidence intervals)
☐	☒	For null hypothesis testing, the test statistic (e.g. F, t, r) with confidence intervals, effect sizes, degrees of freedom and P value noted<br>*Give P values as exact values whenever suitable.*
☒	☐	For Bayesian analysis, information on the choice of priors and Markov chain Monte Carlo settings
☒	☐	For hierarchical and complex designs, identification of the appropriate level for tests and full reporting of outcomes
☐	☒	Estimates of effect sizes (e.g. Cohen's d, Pearson's r), indicating how they were calculated

Our web collection on statistics for biologists contains articles on many of the points above.

Software and code

Policy information about availability of computer code

Data collection	BD FACS Sortware (1.2.0.142)<br>CytExpert SRT (1.1)<br>Cutadapt (3.0)<br>Bowtie2 (2.2.9)<br>samtools (1.10)<br>scDamAndTools (1.0)
Data analysis	R (4.1)<br>scDamAndTools (1.0)<br>MAbIDR R package (V1.0)<br>Signac (1.9)<br>Destiny (3.14)<br>Seurat (4.9.9.9044)<br>https://github.com/KindLab/MAbID

For manuscripts utilizing custom algorithms or software that are central to the research but not yet described in published literature, software must be made available to editors and reviewers. We strongly encourage code deposition in a community repository (e.g. GitHub). See the Nature Portfolio guidelines for submitting code & software for further information.

Data

Policy information about availability of data

All manuscripts must include a data availability statement. This statement should provide the following information, where applicable:

- Accession codes, unique identifiers, or web links for publicly available datasets
- A description of any restrictions on data availability
- For clinical datasets or third party data, please ensure that the statement adheres to our policy

All relevant data supporting the findings of this study are available within the article and its supplementary information files. All raw sequencing data and processed files are made available on the GEO database under accession GSE218476. Any other datasets mentioned in the manuscript were generated using the computational protocols described in the methods.

Public ENCODE datasets: ENCFF001SWK, ENCFF002CKI, ENCFF002CKJ, ENCFF002CKK, ENCFF002CKN, ENCFF002CKY, ENCFF002CUS, ENCFF002CTX, ENCFF002CUU, ENCFF002CUN, ENCFF010PHG, ENCFF312LYO, ENCFF444SGK, ENCFF689TMV, ENCFF745HXR, ENCFF827GEM, ENCFF834YLI, ENCFF401KET, ENCFF055NNT
Public CUT&Tag, MulTI-Tag, and NTT-seq datasets: GSM4842201, GSM3536514, GSM3536515, GSM3536516, GSM3536518, GSM3536522, GSM4308161
Public 4D nucleome dataset: 4DNFIX4BXSIM
Public sortChIC dataset: GSM5018603

Human research participants

Policy information about studies involving human research participants and Sex and Gender in Research.

Reporting on sex and gender	This study did not include human research participants.
Population characteristics	This study did not include human research participants.
Recruitment	This study did not include human research participants.
Ethics oversight	This study did not include human research participants.

Note that full information on the approval of the study protocol must also be provided in the manuscript.

Field-specific reporting

Please select the one below that is the best fit for your research. If you are not sure, read the appropriate sections before making your selection.

☒ Life sciences ☐ Behavioural & social sciences ☐ Ecological, evolutionary & environmental sciences

For a reference copy of the document with all sections, see nature.com/documents/nr-reporting-summary-flat.pdf

Life sciences study design

All studies must disclose on these points even when the disclosure is negative.

Sample size	Two replicates were used for bulk MAbID experiments. These were collected as biological replicates, in which cells from established cell lines were grown independently for each replicate. Since these replicates show a high concordance (based on Pearson's r statistical test), no further replicates were included. For single-cell MAbID experiments, single biological samples (no biological replicates) were collected to obtain all single-cell measurements as well as the bulk MAbID reference samples. Over 1424 to 1956 single-cell samples were collected per sample type for the in vitro neural differentiation system, as well as 4862 single cell samples for the mouse bone marrow sample (all numbers reported are before quality filtering). Per cell type, the individual single-cell measurements can function as technical replicates, and a minimum of 800 cells per cell type was collected.
Data exclusions	Several antibodies were tested during the development of the method, to establish their functionality within the MAbID protocol. Some of the antibodies tested did not provide sufficient data quality in terms of signal specificity, which was determined upon comparison with publicly available data. This was especially relevant using primary antibody-DNA conjugates, since the quality of the antibody can decrease during the conjugation procedure. These samples were therefore excluded from further analysis and from the dataset presented in this manuscript.
Replication	Two biological replicates were used for each bulk MAbID experiment. The concordance between these replicates was analyzed and confirmed using Pearson's r statistical testing and UMAP vizualizations. For single-cell MAbID experiments, no biological replicates were included, but individual cell measurements per cell type can in principle be considered as technical replicates. Per cell type, the individual single-cell measurements can function as technical replicates, and a minimum of 800 cells per cell type was collected.
Randomization	Biological replicates were collected in seperate experiments, so these reside in different sample groups. All individual epitope-measurements (using different antibodies) per bulk MAbID experiment were collected in one experiment and combined in one library before sequencing. For single-cell MAbID experiments of the in vitro differentiation dataset, samples and cells were annotated based on the time point of harvest (either mESC or early NPC). K562 cells were taken along as a separate group. All samples were treated equally but kept in separate containers

	per cell type. For the single-cell MAbID experiments of the mouse bone marrow dataset, samples and cells were annotated based on the FACS information - cells were gated based on cell-surface markers to select for specific cell types and enrich for these. K562 cells were taken along as a separate group. K562 cells and mouse bone marrow samples were treated equally but kept in separate containers.
Blinding	Since all samples (both different replicates as well as different epitope-measurements) were treated equally during sample collection and subsequent data analysis, it was not required to include blinding of the investigators. Biological replicates were statistcially compared to confirm concordance between replicates and samples were extensively compared to publicly available data to confirm the validity of the data.

Reporting for specific materials, systems and methods

We require information from authors about some types of materials, experimental systems and methods used in many studies. Here, indicate whether each material, system or method listed is relevant to your study. If you are not sure if a list item applies to your research, read the appropriate section before selecting a response.

Materials & experimental systems

n/a	Involved in the study
☐	☒ Antibodies
☐	☒ Eukaryotic cell lines
☒	☐ Palaeontology and archaeology
☐	☒ Animals and other organisms
☒	☐ Clinical data
☒	☐ Dual use research of concern

Methods

n/a	Involved in the study
☒	☐ ChIP-seq
☐	☒ Flow cytometry
☒	☐ MRI-based neuroimaging

Antibodies

Antibodies used

See Supplementary Table 1 for more extensive information on used antibodies. Short description on all antibodies is included below:
- Anti-Lamin B1 antibody - Nuclear Envelope Marker, Abcam, ab16048, Lot numbers: GR3398319-7, GR3369248-1;
- Histone H3K9me2 antibody (pAb), Active Motif, 39041, Lot numbers: 39239, 34718002;
- H3K9me3 Recombinant Rabbit Monoclonal Antibody (RM389), Invitrogen, MA5-33395, Lot numbers: WI3388337, WH3388337;
- Tri-methyl-histone-H3 (Lys27) Rabbit mAb, Cell Signaling Technologies, 9733S, Lot numbers: 16,19;
- Recombinant Anti-Histone H3 (tri methyl K27) antibody [EPR18607] - BSA and Azide free, Abcam, ab222481, Lot numbers: GR3256223-6, GR3256223-1;
- Anti-Trimethyl-Histone H3 (Lys36) antibody, clone RM155, RevMab, 31-1051-00, Lot numbers: T-04-02948;
- Histone H3 trimethyl K36 antibody, In-house by Hiroshi Kimura, CM333;
- H3K4me3 Monoclonal Antibody (G.532.8), Invitrogen, MA5-11199, Lot numbers: WG3341041, WH334779;
- Histone H3 (mono methyl K4) antibody, Abcam, ab8895, Lot numbers:GR3402097-1;
- Recombinant Anti-Histone H3 (acetyl K27) antibody [EP16602], Abcam, ab177178, Lot numbers: GR3202987-6, GR3202987-19;
- Recombinant Anti-RNA polymerase II CTD repeat YSPTSPS (phospho S5) antibody [3E8], Abcam, ab252852, Lot numbers: GR3302510-1, GR33352497-1;
- CTCF Antibody, Diagenode, C15410210, Lot numbers: A2354-0010;
- SUZ12 (D39F6) XP® Rabbit mAb, Cell Signalling Technologies, 3737S, Lot numbers: 8;
- Histone H3 Antibody, Novus Biologicals, NB100-747, Lot numbers: p60919;
- AffiniPure Goat Anti-Rabbit IgG (H+L), Jackson ImmunoResearch, 111-005-144, Lot numbers: 147466;
- AffiniPure Donkey Anti-Rabbit IgG (H+L), Jackson ImmunoResearch, 711-005-152, Lot numbers: 156033;
- AffiniPure Donkey Anti-Mouse IgG (H+L), Jackson ImmunoResearch, 715-005-150, Lot numbers: 155934;
- AffiniPure Donkey Anti-Rat IgG (H+L), Jackson ImmunoResearch, 712-005-150, Lot numbers: 154663;
- AffiniPure Donkey Anti-Sheep IgG (H+L) , Jackson ImmunoResearch, 713-005-147, Lot numbers: 150929;
- Alexa Fluor® 647 anti-mouse Ly-6G/Ly-6C (Gr-1) Antibody, Biolegend, 108418, Lot numbers: B287274;
- Brilliant Violet 421™ anti-mouse CD19 Antibody, Biolegend, 115549, Lot numbers: B328655;
- PE anti-mouse TER-119/Erythroid Cells Antibody, Biolegend, 116208, Lot numbers: B311713;
- APC/Cyanine7 anti-mouse CD3 Antibody, Biolegend, 100222, Lot numbers: B324939;
- Alexa Fluor® 488 anti-mouse NK-1.1 Antibody, Biolegend, 108718, Lot numbers: B316543;
- All antibody-DNA conjugates (as well as antibody-DBCO-PEG4 intermediates, both primary and secondary) were derived from the previously stated antibodies by following the conjugation procedure described in the methods section.

Validation

All antibodies (except for the Histone H3 trimethyl K36 antibody, in-house by Hiroshi Kimura, CM333) are commercially available and have been verified for specificity by the supplier as described on the specification sheets. Antibodies were further selected based on their compatibility with ChIP-seq, CUT&RUN or CUT&Tag methodology, as indicated by the supplier. The histone H3 trimethyl K36 antibody, in-house by Hiroshi Kimura, CM333, was validated by Western Blotting, Immunofluorescence staining and peptide ELISA. Antibodies were validated within the MAbID method by testing at least two concentrations and comparing the resulting data to publicly available datasets. Only antibodies with specific signal enrichment at the expected genomic locations were included and subsequently used at the concentration that resulted in the highest signal-to-noise ratio. Commercial antibodies for BM cell-surface marker stainings were optimized and validated by performing indivual stainings at different concentrations.

Eukaryotic cell lines

Policy information about cell lines and Sex and Gender in Research

Cell line source(s)	The K562 cells were a gift from the van Steensel lab at the Netherlands Cancer Institute in Amsterdam, The Netherlands. K562 cell line is generated from human female lymphoblast cells isolated from the bone marrow of a 53-year-old chronic myelogenous leukemia patient. mESCs (F1ES) are mouse embryonic stem cells resulting from a hybrid cross between Cast/EiJ (paternal) x 129SvJae (maternal) mice. These cells are female and were gifted by the Joost Gribnau lab from the Erasmus Medical Centre at Rotterdam, The Netherlands.
Authentication	Cell lines were not authenticated, but genomic data obtained from these cells matches the expected reference genome, including known varations in karyotype. mESC cells were grown as standard in feeder conditions to maintain pluripotency, which was monitered visually during cell culture.
Mycoplasma contamination	Cell lines were regulary tested for mycoplamsa contamination (every 3 months) and have always tested negative, using both PCR based and ELISA-based approaches.
Commonly misidentified lines (See ICLAC register)	No commonly misidentified lines were used in this study.

Animals and other research organisms

Policy information about studies involving animals; ARRIVE guidelines recommended for reporting animal research, and Sex and Gender in Research

Laboratory animals	For mouse bone marrow isolations, 4 female mice (littermates) with the C57BL/6NCrl genotype were used, all approximately 9 weeks old. The light/dark cycles constitutes of 14 hours light (starting at 5:00) and 10 hours dark (starting at 19:00). The temperature is kept between 20 and 24 degrees Celsius and the humidity is kept between 40 and 65%.
Wild animals	The study did not involve wild animals.
Reporting on sex	All mice used were female, in order to prevent biases related to sex.
Field-collected samples	The study did not involve samples collected from the field.
Ethics oversight	All mice used in this study were bred and maintained in the Hubrecht Institute Animal Facility. Experimental procedures were approved by the Animal Experimentation Committee of the Royal Netherlands Academy of Arts and Sciences and performed according to the guidelines.

Note that full information on the approval of the study protocol must also be provided in the manuscript.

Flow Cytometry

Plots

Confirm that:

☒ The axis labels state the marker and fluorochrome used (e.g. CD4-FITC).

☒ The axis scales are clearly visible. Include numbers along axes only for bottom left plot of group (a 'group' is an analysis of identical markers).

☒ All plots are contour plots with outliers or pseudocolor plots.

☒ A numerical value for number of cells or percentage (with statistics) is provided.

Methodology

Sample preparation	To isolate mouse bone marrow cells (BM), the tibia and femur bones from the hindlegs were removed. The top of the bone was removed and the marrow was flushed out using a syringe with HBSS buffer (Gibco, 14025092). Cells were isolated from the marrow by pipetting up and down several times and poured through a 70 µm cell strainer (Greiner, 542070) before diluting in 25 mL HBSS buffer. Cells were centrifuged for 10 minutes at 300 g at 4 °C. Supernatant was removed, cells were resuspended in 10 mL PBS and centrifuged at 500g for 5 min. After removing the supernatant, cells were counted and fixated (ethanol fixation) before freezing until further use - see methods for further details. Before sorting, BM cells were thawed on ice and washed twice in Wash buffer 1 (20mM HEPES pH 7.5,, 150 mM NaCl, 66.6 µg/mL Spermidine, 1X cOmplete™ protease inhibitor cocktail, 0.05% Tween20 (Sigma, P9416), 2mM EDTA) before antibody incubation. Samples were incubated with primary antibody-DNA conjugates overnight and several washing steps (with Wash Buffer 2, 20mM HEPES pH 7.5, 150 mM NaCl, 66.6 µg/mL Spermidine, 1X cOmplete™ protease inhibitor cocktail, 0.05% Tween20) were done afterwards to remove unbound antibody. Directly following primary antibody-DNA conjugate incubation, BM cells were washed once with Wash Buffer 2 and resuspended in 400 µL Wash Buffer 2 containing 5% Blocking Rat Serum (Sigma, R9759) per 1 million cells. Cells were incubated with commercial antibody-fluorophore conjugates against specific BM surface markers of Granulocytes, B cells, T cells, Erythroblasts and NK cells (see Supplementary Table 1 for antibodies and concentrations). Incubations were performed for 30 minutes at 4 °C on a tube roller. Samples were kept in the dark from this point onwards. Finally, cells were

washed once with Wash Buffer 2 and resuspended in 1 mL Wash Buffer 2 before proceeding to FACS sorting.

Instrument	A Beckman Coulter CytoFLEX SRT Benchtop Cell Sorter was used for all mouse bone marrow sorts. For other FACS sorts of K562, mESC or early NPCs cells, BD FACSJazz™ Cell Sorter and BD Influx™ Cell Sorter machines were used.
Software	Beckman Coulter software was used for all data acquirements.<br>BD FACS Sortware (1.2.0.142)<br>CytExpert SRT (1.1)
Cell population abundance	Single-cell samples were sorted and directly processed in the scMAbID protocol. Proper enrichment and purity of cell types was confirmed by assessing the quality metrics of the scMAbID dataset.
Gating strategy	The gating strategy is shown in Extended Data Figure 8a. Cells were first gated generally on FSC and SSC values (gates P1 to P2) to obtain high quality single-cell samples. From P2, Granulocytes were gated based on the GR1 - AF647 [660]-660-10-A value. The remaining cells were gated through P3. From P3, B cells were gated based on the CD19 - BV421 [405]-450-45-A value and NK cells were gated on the NK1 - AF488 [488]-525-40-A value. Remaining cells were gated in P4. From P4, T cells were selected gated on the CD3 - APC-Cy7 [660]-780-60-A value. Remaining cells were gated in P5. FInally, from P5 Erythroblasts were gated based on the TER119 - PE [561]-585-42-A. Boundaries in all cases were selected to obtain the cells with the highest values, ideally with some seperation from the non-gated cells.

☒ Tick this box to confirm that a figure exemplifying the gating strategy is provided in the Supplementary Information.
