# Supplementary figures and images for: Combinatorial single-cell profiling of major chromatin types with MAbID
Source: Nat Methods. 2023 Dec 4;21(1):72–82. doi: 10.1038/s41592-023-02090-9 (PMC10776404; doi:10.1038/s41592-023-02090-9)

a

Extended Data Figure 1a

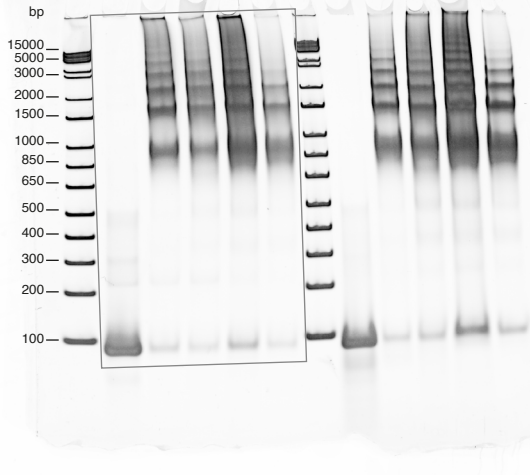

b

Extended Data Figure 5a

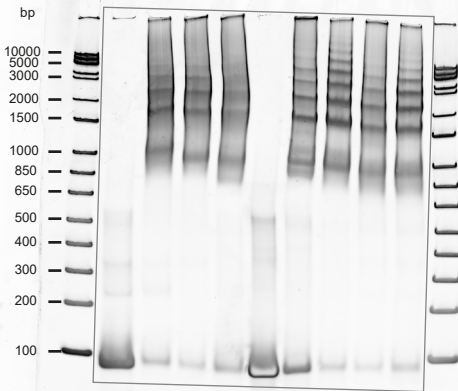

Supplement: Supplementary file 5 — Unprocessed gels for Extended Data Figs. 1a and 5a. [file 41592_2023_2090_MOESM5_ESM.pdf]
